# I Want My Bullet Back: Percutaneous Endovascular Removal of a Right Atrial Foreign Body

**DOI:** 10.7759/cureus.3367

**Published:** 2018-09-26

**Authors:** Taylor S Harmon, Todd Loper, Jerry Matteo

**Affiliations:** 1 Interventional Radiology, The University of Texas Medical Branch, Galveston, USA; 2 Interventional Radiology, Flagler Hospital, St. Augustine, USA; 3 Interventional Radiology, University of Florida College of Medicine, Jacksonville, USA

**Keywords:** endovascular, gunshot trauma, firearm, snare, homicide, bullet embolus, ivc, right atrium, interventional radiology, internal jugular vein

## Abstract

The incidence and prevalence of firearm-related homicide in the United States make headlines daily. As a result, an epidemic of penetrating injuries is on the rise. Specifically, foreign bodies such as bullets and shrapnel are usually left inside the human body due to penetrating injuries, unless they are in close proximity to vital structures. We present a case of a bullet within the right atrial chamber of the heart, which was successfully removed by a minimally invasive endovascular approach.

## Introduction

According to the Organization for Economic Cooperation and Development, homicide rates are seven times higher in the United States compared to other high-income countries, driven by a firearm-related homicide rate that is 25.2 times higher than the next leading country [1]. In 2016, there were 37,863 total firearm-related homicides, equating to just over 100 deaths per day and more than four firearm-related deaths every hour [2]. In certain demographic populations, such as between the ages of 15-34, the firearm-related homicide rates are ten times higher, creating an obvious disparity of fire-arm related violence between other age groups [3]. In this same age group alone, the firearm-related homicide rate per 100,000 was eight to nine in 2012, and has been increasing with the most recent crude rate of firearm-related homicide to be 17.34 per 100,000 in 2016 [2-3].

As gun violence has become an expanding and alarming issue in the United States, the management of gunshot-induced trauma has also become a common scenario for physicians. Likewise, the increasing amount of gunshot-induced trauma has allowed for unique case-dependent scenarios, leading to specifically tailored management of these patients as dictated by their clinical presentations. The following case will demonstrate the minimally invasive interventional management of a gunshot-induced trauma that was necessary for avoiding damage to the patient’s vital surrounding structures.

## Case presentation

**Figure 1:**
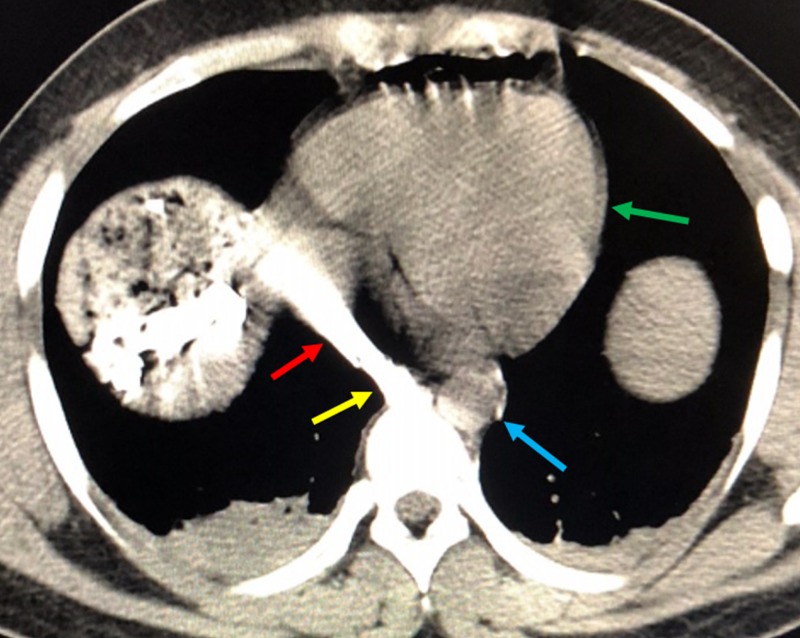
Initial Non-Contrast Axial Computed Tomography of the Chest The bullet is seen in the inferior vena cava (yellow arrow). There is significant streak artifact from the metallic shrapnel (red arrow) adjacent to the intact aorta (blue arrow). The green arrow delineates the heart.

We present the case of a 27-year-old African American male who was shot with a 9 mm handgun during an attempted home invasion and burglary. An initial axial non-contrast computed tomography (CT) of the chest and abdomen was done that showed a bullet found anterior to the abdominal midline. The bullet had ricocheted off of the T12 vertebral body and penetrated the inferior vena cava (IVC) (Figure 1).

**Figure 2:**
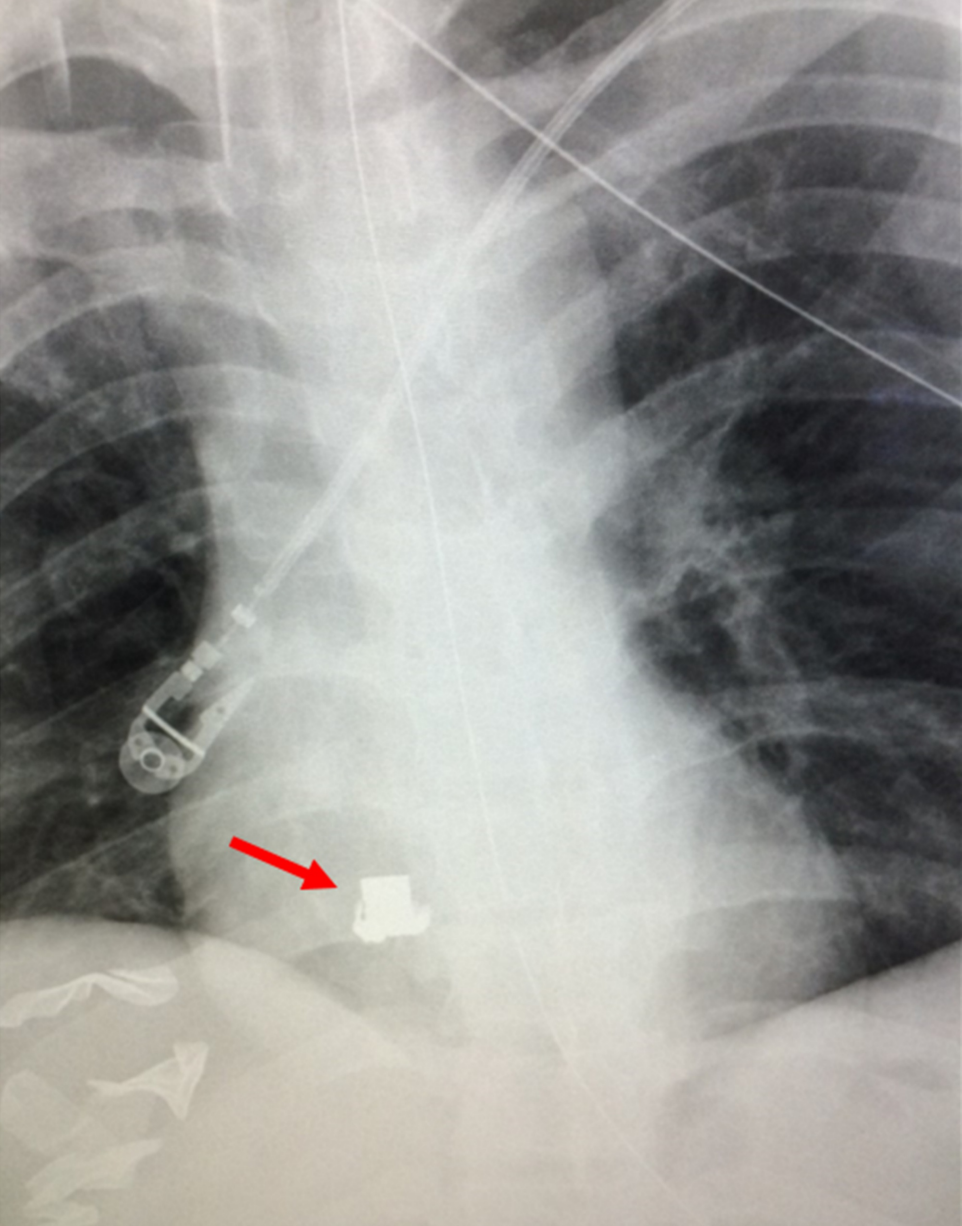
Post-Surgical Chest Radiograph A post-surgical chest radiograph is shown after surgical clamping of the patient's inferior vena cava. A radiopaque bullet is shown overlying the right heart (red arrow).

The patient was then taken to the operating room for emergency exploratory surgery. The IVC was clamped and the patient was intubated and placed on life support. A follow-up chest radiograph 48 hours later demonstrated a 9 mm bullet that was seen in a different location projecting over the right atrium of the heart. This differs from the initial CT scan (Figure 2).

**Figure 3:**
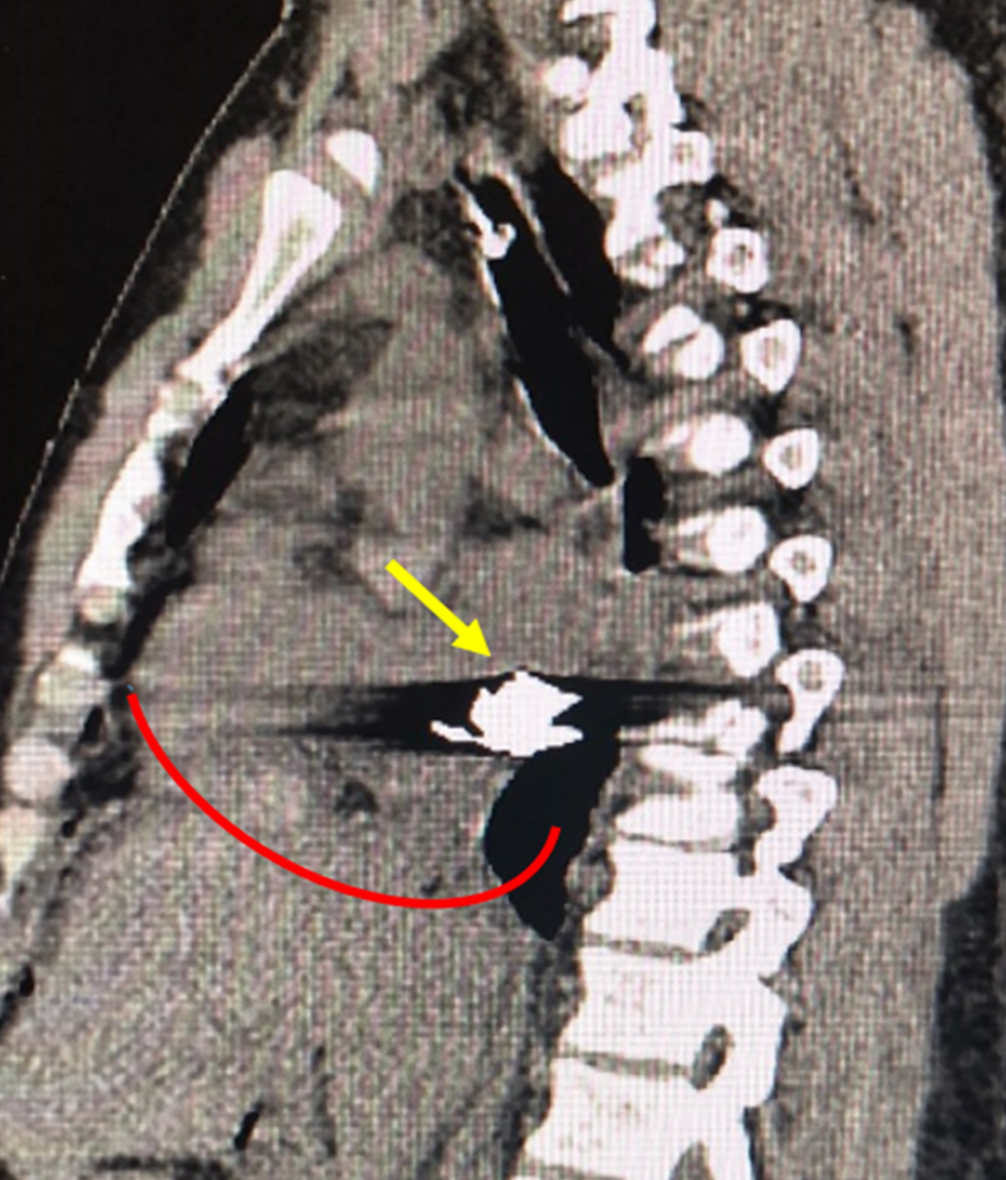
Post-Surgical Confirmatory Non-Contrast Sagittal Computed Tomography of the Thorax A post-surgical non-contrast sagittal computed tomography of the thorax confirms the anatomic and spatial location of a bullet embolus (yellow arrow) that migrated from the patient's inferior vena cava into the right atrium. The inferior heart border is delineated by the curved red line.

The two-dimensional anterior-posterior view of the chest radiograph conducted could not confirm the spatial orientation or anatomic location of the bullet. Therefore, a non-contrast sagittal CT confirmed that the bullet had migrated from the IVC into the right atrium of the heart (Figure 3).

**Figure 4:**
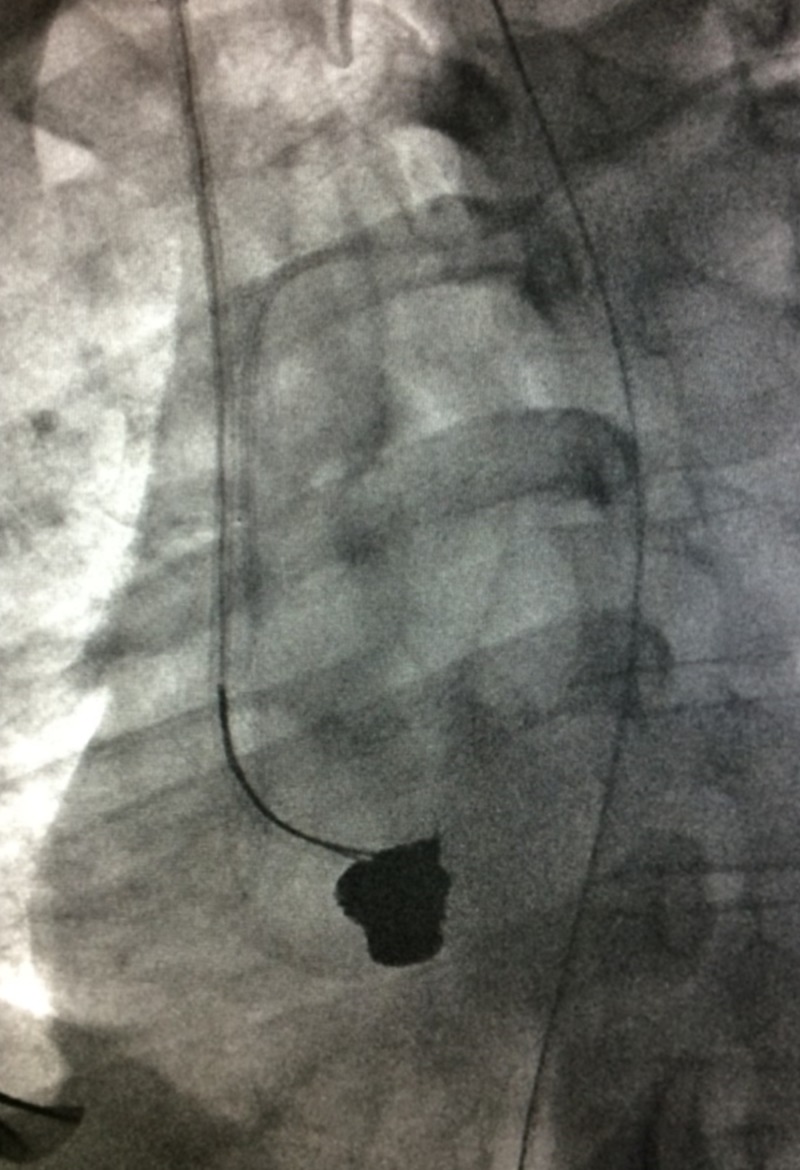
Snare Entrapment of Bullet Embolus on Fluoroscopy A fluoroscopic view of a snare that was used to lasso a bullet embolus against a guiding sheath.

The concerns in the further management of the trauma and cardiothoracic surgery teams included the possibility that the bullet could cause myocardium perforation, pericardial injury, or further migration into the patient’s pulmonary artery. Interventional radiology was consulted for the possibility of performing an endovascular extraction of the bullet through the right internal jugular vein (IJV). The interventional radiology team agreed to proceed with the extraction, and the patient was brought to special procedures. The patient’s right neck was prepared and draped in a sterile fashion to initiate venous access into the IJV. Using ultrasound guidance, access was obtained into the right IJV using a micropuncture kit. Through the micropuncture sheath, a Benson wire was advanced into the right IJV, superior vena cava (SVC), and ultimately the IVC. A 10 cm by 11 cm bright tip sheath was then advanced over the Benson wire, into the right IJV. A 12 mm by 20 mm snare device was advanced over a 6 French sheath into the right atrium. Ultimately, the bullet was snared with tension placed on the ensnare device, which lassoed the bullet against the guiding sheath (Figure 4).

**Figure 5:**
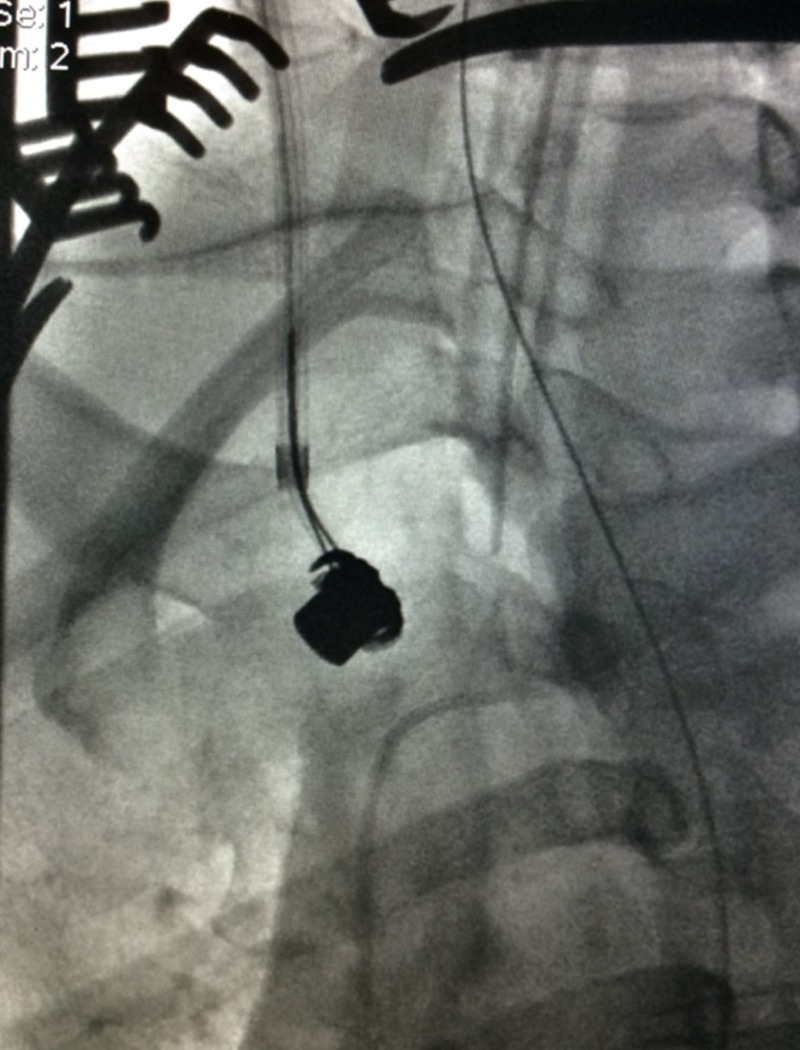
Ascent of Snared Bullet Embolus on Fluoroscopy A fluoroscopic view shows the ascent of a bullet embolus out of the patient's right atrium. The serrated edges made it difficult to successfully retrieve the bullet.

The serrated edges of the bullet made the removal difficult once snared, and numerous attempts to reposition the bullet were made to allow for preservation of the surrounding structures. The bullet was then retrieved once it was certain to have a clear path for removal out of the right atrium, SVC, and right IJV (Figure 5).

**Figure 6:**
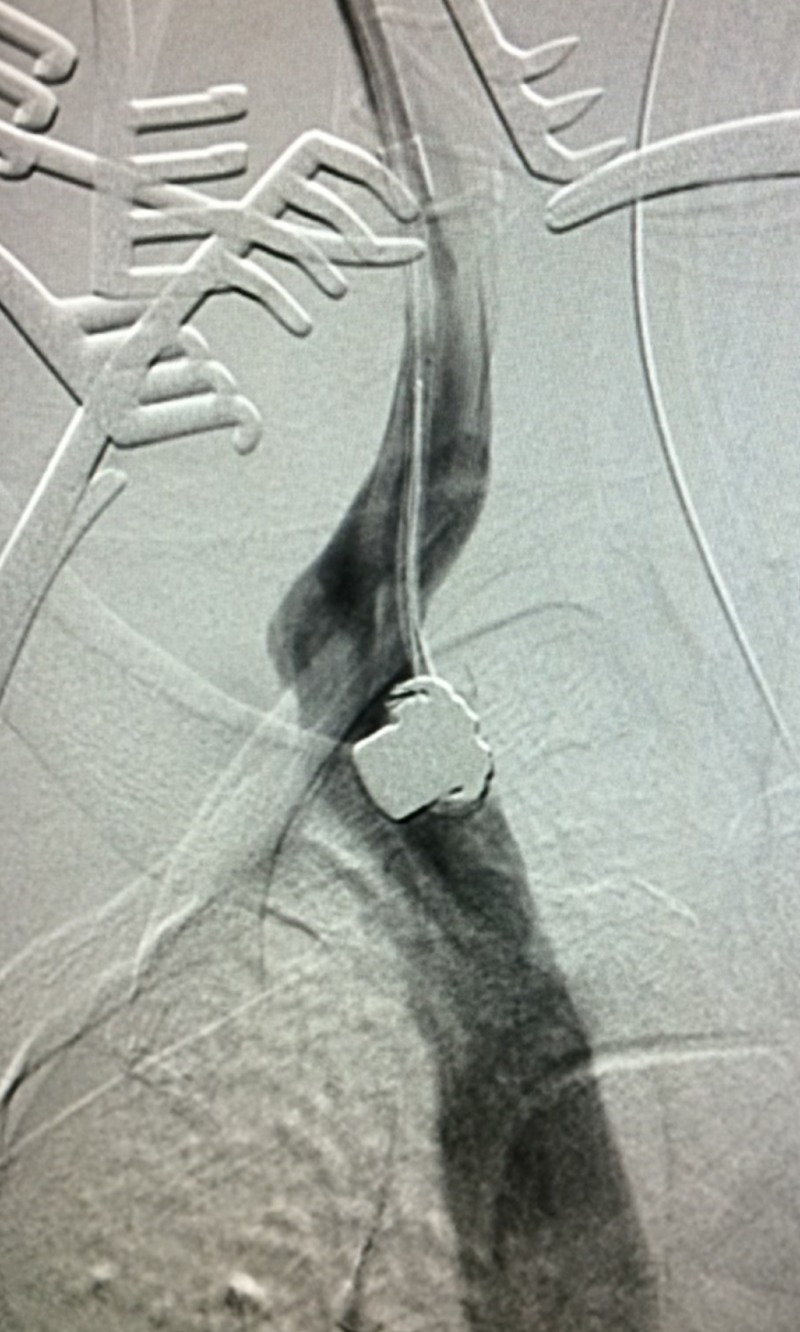
Contrast Venogram Confirming Location of Snared Bullet Embolus A contrast venogram was conducted to confirm the location of the bullet embolus, in order to assist in preparation for percutaneous retrieval.

A venogram of the proximal-most aspect of the right IJV as shown in Figure 6 was conducted once above the level of the clavicles to evaluate vascular injury, extravasation, or hemorrhage.

**Figure 7:**
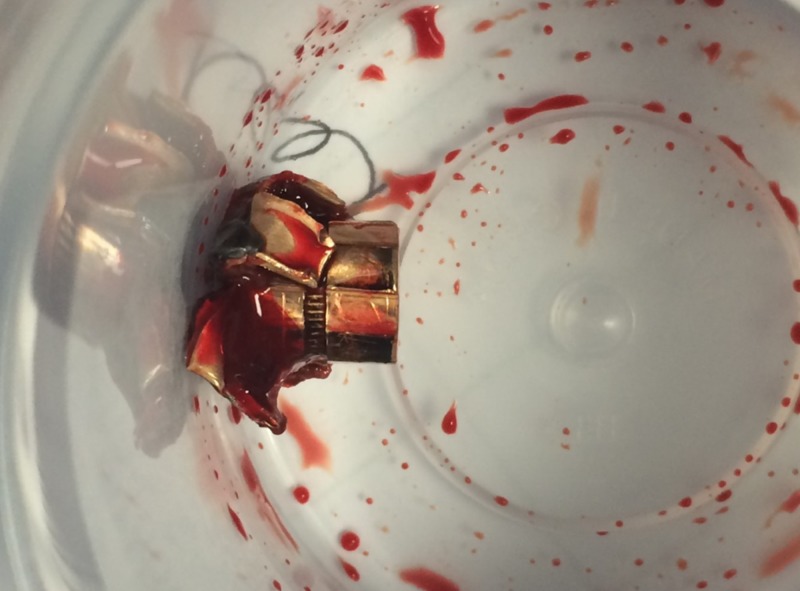
Successful Percutaneous Retrieval of Bullet Embolus The bullet is shown after successful percutaneous interventional retrieval with surgical assistance.

Surgical incision and cut down of the right IJV inferior to the percutaneous access site was conducted in order to provide a controlled exit site for the bullet. Vessel loops were placed around the right IJV superior and inferior to the incision. After the surgical incision, control of bleeding, and evacuation of blood, the bullet was identified (Figure 7).

The snare device and vascular sheath were removed, and a multi-layer surgical closure of the right IJV was performed.

## Discussion

The rise of firearm-related homicide in the United States has led to the increased management of gunshot wound traumas. Furthermore, the patient presented represents an age group population for which firearm-related injury is highly prevalent. From patient-to-patient, gunshot trauma is managed based on the case scenario. It is not uncommon for penetrating injury to be managed by surgical intervention, especially if the patient is hemodynamically unstable [4]. However, surgical intervention of a foreign body (bullet) could also be contraindicated, as it could lead to further damage of the patient’s surrounding structures. Other factors to consider when managing a gunshot trauma are the area of anatomic penetration, location of surrounding structures, and damage to vital organs.

There have been documented cases of peripheral bullet embolization from the extremities to the right heart that have resulted from penetrating gunshot wound traumas [5]. The literature suggests that bullet embolization to the right heart can be managed based on the patient's symptoms, or risk for further embolization into the pulmonary artery [6]. However, in either case, conservative management could pose a future risk for embolization, and surgical interventional could damage the surrounding myocardial tissue (as was the case in our patient). The solution, in either case, would be the interventional extraction of the foreign body (bullet) to defeat both the likelihood of further embolization, or risk of unnecessary invasive surgical intervention.

In this case, an intuitive surgical intervention was initially indicated for gunshot trauma, leading to the clamping of the IVC. The bullet was not indicated for removal, as doing so would have caused further damage to the surrounding structures and possible decompensation of the patient. However, the post-surgical chest x-ray and CT confirmed that the bullet had migrated from the patient’s IVC to the right atrium, requiring further discussion of management from the surgical teams. In an attempt to remove the bullet embolus, interventional radiology used a method for extraction which also preserved the patient’s myocardial tissue and prevented any other damage to the surrounding structures.

## Conclusions

Penetrating gunshot trauma is on the rise with the increasing amount of firearm-related homicide in the United States. Gunshot-related trauma affects certain age groups more prolifically than others. This case demonstrates a successful minimally invasive method for extraction of a bullet embolism that migrated from the patient’s IVC to the right atrium. Snare extraction of a bullet embolus found in the right atrium preserves the patient’s myocardial tissue and surrounding structures by avoiding invasive surgical intervention.
